# Design and evaluation of psychometric properties of cigarette smoking tendency questionnaire for female adolescents (CTQFA)

**DOI:** 10.1186/s12889-021-11784-8

**Published:** 2021-09-25

**Authors:** Alireza Jafari, Nooshin Peyman, Mahdi Gholian-Aval, Mehrsadat Mahdizadeh, Hadi Tehrani

**Affiliations:** 1 Department of Health Education and Health Promotion, School of Health, Social Development and Health Promotion Research Center, Gonabad University of Medical Sciences, Gonabad, Iran; 2 Student Research Committee, Mashhad University of Medical Sciences, Mashhad, Iran; 3 Department of Health Education and Health Promotion, Social Determinants of Health Research Center, Mashhad University of Medical Sciences, Mashhad, Iran

**Keywords:** Validity and reliability, Psychometric, Cigarette smoking, Tobacco

## Abstract

**Background:**

The tendency of women to smoke has increased in recent years and the prevalence of smoking among women is increasing. The purpose of this study was to design and evaluation the psychometric properties of the smoking tendency questionnaire for Iranian female adolescents.

**Methods:**

This cross-sectional study was performed on 604 female adolescents in Iran in 2021. The bank of questions was designed based on the qualitative study concepts and review of the literature. To perform the psychometric evaluation, steps such as face validity (qualitative), content validity (qualitative and quantitative) and construct validity (confirmatory factor analysis) were performed. The reliability of the instrument was assessed using McDonald’s omega coefficient and Cronbach’s alpha coefficient.

**Results:**

Based on the results of psychometrics (face, content, and construct validity), the number of questions was reduced from 102 to 52, and 50 questions were removed. Finally, a questionnaire with 52 questions and 5 subscales of the tendency to experience smoking (14 items), re-experience smoking (8 items), cigarette dependence (9 items), intention to quit smoking (9 items), and smoking cessation (12 items) was approved. The content validity ratio (CVR) and content validity index (CVI) for all questions were 0.770 and 0.938, respectively. The Cronbach’s alpha and McDonald’s omega coefficients for all questions were 0.903 and 0.904, respectively.

**Conclusion:**

Based on the results of this questionnaire, 52 questions, and 5 subscales can be used to assess the tendency of female adolescents to cigarette smoking.

## Background

Tobacco use is one of the major public health issues, concerns and threats to global health [1, 2]. Tobacco smoking causes the death of 8 million people a year and is the leading cause of preventable death in the world [3]. Smoking is considered as one of the risk factors, increasing the overall burden of diseases in the world, especially those related to chronic diseases and non-communicable diseases (such as cardiovascular, respiratory system, cancer and stroke) [4].

There are more than 1.1 billion smokers and at least 367 million smokeless tobacco users worldwide [3]. Tobacco smoking has been identified as a negative health factor including cancer, vascular, and respiratory diseases [5], and has been responsible for approximately 11.5% of all deaths in 2015 [6]. Approximately 1 out of 25 people (187 to 280 million people) undergo major surgery to treat diseases or injuries caused by smoking every year [7, 8]. The prevalence of cigarette smoking has been steadily increasing in developing countries over the past 20 years and declining in developed countries [9]. Based on the research results, 70% of smoking in the world is in developing countries [10]. Among smoker, 85% cigarettes smoking, and one of the top 10 causes of death in the world is related to smoking [3, 10].

Based on the World Health Organization (WHO) report, 8.5% of girls have used tobacco, of which 5.5 and 4.8% are currently using tobacco and cigarette respectively [3]. The results of a meta-analysis study conducted in 2020 showed that the current and ever prevalence of cigarette smoking in Iranian adolescent girls were 6 and 12%, respectively [11]. The prevalence of cigarette smoking in women increases the risk of cardiovascular disease, stroke, shortness of breath, cervical cancer, and breast cancer [12–15]. Also, smoking in pregnant women can causes problems for mother and fetus, such as stillbirth, fetal measurements, neonatal death, perinatal death, placental abruption, miscarriage, preterm birth, premature pulmonary aging, and chronic obstructive pulmonary disease [16–18].

Based on the results of various studies, reasons such as smoking parents, smoking by family members, alcohol consumption, low self-esteem, curiosity, low parental education, parental divorce, or living with a parent, lack of appropriate choices to reduce stress, low-income family, belongs to the smokers group, have a friend who smokes, peer group pressure, tobacco company advertisements, smoking by celebrities, smoking attraction, and positive attitude to smoking are effective in adolescents’ tendency to cigarette smoking [19–26].

The smoking prevalence of Iranian women has increased in recent years and is becoming a normal social phenomenon. Previously, smoking was regarded as a taboo for women in Iranian society [11, 27]. Based on the literature, various questionnaires related to smoking have been designed and implemented. In Iran and other countries, there was not any tool that comprehensively examines all aspects of adolescent tendency to smoking, especially in adolescent girls. The available questionnaires only examined some aspects of smoking, such as quit Smoking, attitude toward smoking, prevention of smoking, intention to smoking, and dependence on smoking [28–34]. However, since this phenomenon is new in Iran, there have been few studies in this field and in females. Also, a suitable tool to examine the reasons for adolescent girls’ tendency to smoke, not observed.

Due to the increasing smoking rate among female adolescents and the lack of many tools, it is necessary to design a reliable and valid tool in this field to assess the current situation and evaluate intervention programs. Therefore, the purpose of this study was to design and evaluate the psychometric properties of the smoking tendency questionnaire for Iranian female adolescents.

## Methods

This cross-sectional study was conducted among 604 female adolescents in Mashhad, Iran in 2021.

### Sample size

Based on a confidence level of 0.95%, a proportion of 0.12, an accuracy of 0.03, and a sample loss of 25%, the sample size was estimated to be 604 subjects [11].

### Sampling

Participants were selected by the multi-stage method. At first, Mashhad city was divided into four parts: north, south, east, and west. Then, four girls’ high schools were selected from each region as a cluster (*n* = 16 schools). Then, about 38 female adolescents from each school entered the study by simple random sampling. Inclusion criteria were a female student in high school (tenth, eleventh and twelfth grades), resident of Mashhad, consent to participate in the study, fill out written informed consent from by students and their parents. Exclusion criteria also included questionnaires with distorted and incomplete.

### Instruments

In this study, two questionnaires of demographic and the tendency of female adolescents to cigarette smoking (CTQFA) were used for data gathering.

#### The demographic questionnaire

This section was surveyed with questions such as education grad, age, education level of parents, and employment status of parents.

#### Cigarette smoking tendency questionnaire for female adolescents (CTQFA)

This questionnaire has 52 questions and 5 subscales of the tendency to experience smoking (14 items), re-experience smoking (8 items), cigarette dependence (9 items), intention to quit smoking (9 items) and smoking cessation (12 items). All questions were measured on a 5-point Likert scale (strongly disagree = 1, disagree = 2, neither agree nor disagree = 3, agree = 4, and strongly agree = 5).

### Design of instrument

#### Qualitative stage

At this stage, a grounded theory study was conducted among adolescent girls who smoked. For the interviews, we went to parks, places where tobacco is used, etc., and the interviews continued until the data was saturated. Data were collected by unstructured and in-depth interviews with adolescent female smokers in different stages of smoking (*n* = 23), parents of female smokers and non-smokers (n = 2), clinical psychologist (n = 2), sociologist (*n* = 1), and tobacco seller (n = 1). The code extraction step was performed by MAXQADA software version 10. The analysis process was based on the Corbin & Strauss 2008, which is divided into three stages of open, axial and selective coding [35].

#### Quantitative stage

At this stage of the research, a series of questions were designed according to the concept of qualitative research and literature review. For psychometric evaluation, steps such as face validity (qualitative), content validity (qualitative and quantitative) and construct validity (confirmatory factor analysis) were performed. The reliability of the instrument was assessed using McDonald’s omega coefficient and Cronbach’s alpha coefficient. The details of these steps are as follows.

### Validity

#### Face validity

To assess the qualitative face validity, a questionnaire was sent to 6 experts to examine the questionnaire in terms of the desirability of expressions in terms of clarity (use of simple and understandable words), use of common language (avoidance of using technical and specialized words). Then, based on the comments received, the necessary amendments were made to the questionnaire. Also, an interview was conducted with the target group to discover the possibility of difficulties in understanding phrases and words, proper matching and relevance of items, ambiguity and inaccurate interpretation of phrases, or insufficient meaning of words.

#### Content validity

To determine the qualitative content validity, the questionnaire was provided to 12 experts and specialists in the field of health education and health promotion and clinical psychology to evaluate the items such as grammar, use of appropriate words, the importance of items, placement of items in the appropriate part, and the time required to complete the tool. To evaluate quantitative content validity, two methods were used, content validity ratio (CVR) and content validity index (CVI) [36].

##### CVR

The questionnaire was provided to 12 experts/specialists in the field of health education and health promotion and clinical psychology and who were asked to rate each item of the tool with three levels “essential”, “useful but it is not necessary”, and “it is not necessary”. The answers were calculated based on the following formula. To calculate the CVR, the Lawshe formula was used (in this formula, n_E_ is the number of experts selected item of “essential” and N is the number of total experts) [37].

$$ CVR=\frac{n_E-(N2)\kern0.5em }{N/2} $$

##### CVI

In this section, experts were asked to comment on each item of the tool based on the following three criteria (relevance or specificity, simplicity and fluency, clarity or transparency) on a four-point Likert scale. The CVI was calculated using the following formula. A CVI score of 0.78 and above is considered acceptable [38].

$$ CVI=\frac{Nu\mathrm{m} ber\  of\ \mathrm{experts}\  selected\ items\ of\ 3\  and\ 4}{Total\ number\ of\ \mathrm{experts}\ } $$

#### Construct validity

Confirmatory factor analysis (CFA) was used to evaluate the construct validity. Before performing the CFA, the outliers and the normality of data were examined. The outliers were examined with the help of Mahalanobis distance statistics and were deleted if necessary. Skewness and kurtosis were used to evaluate the data normality. Also, the maximum likelihood method was used to estimate the parameters. CFA was conducted using AMOS software version 24, and questions with poor regression coefficient (factor loading) were removed from the questionnaire. The model was evaluated using fit indices of chi-square ratio to the degree of freedom (× 2/df < 5); root means the square error of approximation (RMSEA ≤0.08); root means square residual (RMR ≤ 0.08); goodness of fit index (GFI > 0.9); parsimony comparative fit index (PCFI > 0.5); parsimonious normed fit index (PNFI > 0.5); parsimony goodness-of-fit index (PGFI > 0.5); and comparative fit index (CFI > 0.9) [39–42].

### Reliability

In this study, two methods of McDonald’s omega coefficient and Cronbach’s alpha coefficient were used to evaluate the reliability of the questionnaire. The software’s of SPSS_v22_ and JASP (Version 0.11.1) were used to calculate the amount of Cronbach’s alpha coefficient and McDonald’s omega coefficient. Results showed that McDonald’s omega coefficient provides a more accurate reliability coefficient than Cronbach’s alpha [43]. Reliability coefficient value exceeding 0.70 is considered acceptable [44].

## Results

### The characteristics of the participants

In the present study, the mean (**±** standard deviation) age of female adolescents was 16.47 (**±**1.06). Most students (*n* = 271, 44.9%) were in the twelfth grade. The level of education of most fathers (*n* = 310, 51.3%) and mothers (*n* = 249, 41.3%) was associate or bachelor’s degree. Forty-two percent (*n* = 255) of the adolescent fathers were employed and most of the adolescent mothers were housewives (*n* = 342, 56.7%) (Table 1).

**Table 1:** Frequency distribution of demographic characteristics (*n* = 604)

Variables		N	%
**Grade**	Tenth	171	28.3
	Eleventh	162	26.8
	Twelfth	271	44.9
**Father’s education level**	Illiterate	16	2.6
	Elementary	15	2.5
	Middle school	37	6.1
	High school or diploma	142	23.5
	Associate or Bachelor’s Degree	310	51.3
	Master’s degree or High degree	84	13.9
**Mother’s education level**	Illiterate	33	5.5
	Elementary	24	4
	Middle school	35	5.8
	High school or diploma	220	36.5
	Associate or Bachelor’s Degree	249	41.3
	Master’s degree or High degree	42	7
**Father’s occupation**	Unemployed	25	4.1
	Self-employed	217	36
	Labor	50	8.3
	Employed	255	42.3
	Retired	56	9.3
**Mother’s occupation**	Housewife	342	56.7
	Self-employed	62	10.3
	Employed	176	29.2
	Retired	23	3.8

### Qualitative phase

**Fig. 1:**
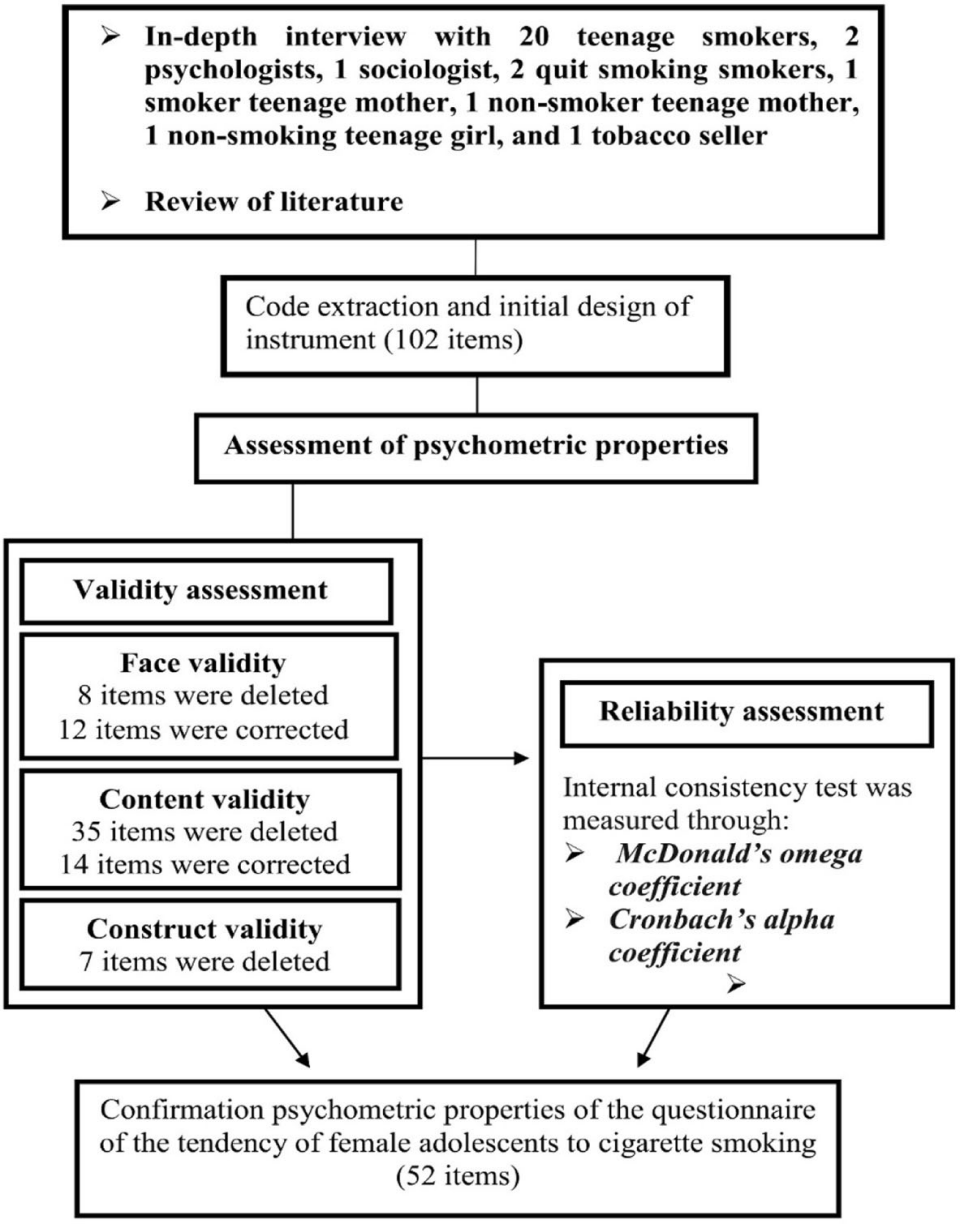
Schematic process of the reduction of the items of questionnaire

The qualitative phase was based on the grounded theory method and in-depth unstructured interviews. Factors influencing the tendency of female adolescents were identified, which included 5 stages of the tendency to experience smoking, re-experience smoking, cigarette dependence, intention to quit smoking, and smoking cessation. In this phase, 102 questions were designed based on the qualitative phase and review of the literature (Fig. 1).

### Quantitative phase

In this phase, the questionnaire was examined in terms of face validity, content validity, and construct validity.

#### Face validity

Based on the opinion of experts in qualitative face validity, 30 questions were corrected. In the quantitative face validity section, 8 questions were deleted and 12 questions were corrected. Finally, in this section, 94 questions remained and entered the content validity stage (Fig. 1).

#### Content validity

Based on the opinion of experts, 19 questions were corrected in qualitative content validity. In the quantitative content validity section, 35 questions were deleted and 14 questions were corrected. Finally, in this section, 59 questions remained and entered the construct validity stage (Fig. 1). The CVR and CVI for all questions were 0.770 and 0.938, respectively.

#### Construct validity

**Fig. 2:**
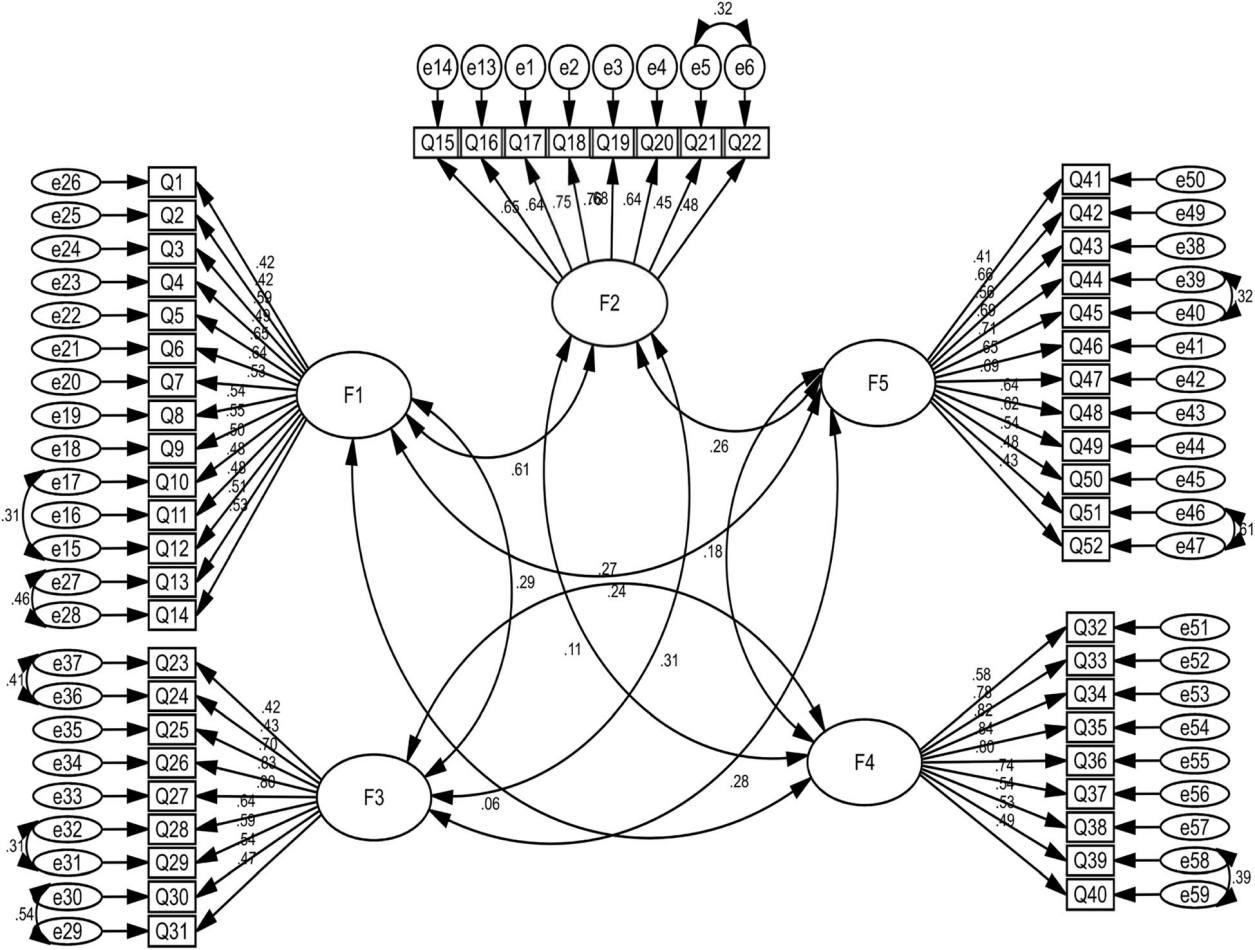
Standardized parameter estimates for the factor structure of the questionnaire (CTQFA); (F1: Tendency to experience smoking; F2: Re-experience smoking; F3: Cigarette dependence; F4: Intention to quit smoking; F5: Smoking cessation)

The CFA was used to evaluate the construct validity of the questionnaire. In this stage, questions with low regression coefficients (factor loading) were deleted to achieve an acceptable model. Based on the results of the CFA analysis, the CR value per question was higher than 1.96, and with a significance level of < 0.001. The goodness of fit the model for five subscales was acceptable (X^2^ = 3686.168, df = 1255, X^2^/df = 2.93, RMSEA = 0.057, RMR = 0.031, GFI = 0.901, PCFI = 0.772, PNFI = 0.706, PGFI = 0.730, CFI = 0.901). In this section, 7 questions were deleted (Fig. 1). Finally, the questionnaire with 52 questions and 5 subscales of the tendency to experience smoking (14 items), re-experience smoking (8 items), cigarette dependence (9 items), intention to quit smoking (9 items), and smoking cessation (12 items) was approved (Fig. 2, Table 2).

**Table 2:** Factor loadings of the questionnaire of factors affecting the tendency of female adolescents to cigarette smoking (CTQFA) in the CFA stage

Subscales	Items	Factor loadings	CVR	CVI
**F1: Tendency to experience smoking**	Q1: Curiosity is very effective for the tendency to smoke.	0.422	1	0.93
	Q2: Smoking of cigarettes and tobacco like a hookah by a family member (father, mother, brother, etc.) encourages smoking.	0.423	0.6	1
	Q3: Experiencing unpleasant events in life (parental differences, parental divorce, suicide, sexual abuse, etc.) can lead to a tendency to smoke.	0.591	0.8	0.86
	Q4: Smoking friends are encouraged to smoke.	0.490	0.8	1
	Q5: Approval from friends for the experience of smoking can be important.	0.648	0.8	0.93
	Q6: The favorable opinion of smokers (friends, colleagues and relatives) about the benefits of smoking causes adolescents tendency to smoke.	0.641	0.8	0.93
	Q7: Parents’ strictness or inattention to various issues is important in the adolescents’ tendency to smoke.	0.534	1	0.90
	Q8: Having religious beliefs can prevent the tendency to smoke.	0.539	0.8	0.83
	Q9: School education programs in the field of smoking (skills of saying no, controlling anger, etc.) reduce the tendency to smoke.	0.548	0.6	0.96
	Q10: Communication networks such as Telegram, Instagram, etc. are effective in encouraging adolescents to smoke.	0.500	1	1
	Q11: Parents’ advice and counseling is a barrier to smoking.	0.482	1	1
	Q12: Smoking of celebrities (artists, athletes, etc.) or people who are important to adolescents (such as friends, relatives, etc.) is an incentive to experience smoking.	0.477	0.8	0.96
	Q13: Gender dissatisfaction, like dissatisfaction with being a girl, is important in the tendency to smoke.	0.510	0.8	1
	Q14: Attractive appearance and beautiful packaging of cigarettes are important in the tendency to smoke.	0.528	0.6	1
**F2: Re-experience smoking**	Q15: Compared with other tobaccos, cigarettes are easy to use and easy to obtain, leading to continuous smoking.	0.652	0.6	0.96
	Q16: Proving yourself to friends to gain social popularity is an effective factor in continuing to smoke	0.644	0.8	0.93
	Q17: The normalization of smoking by women in society is a stimulus for continued smoking.	0.750	0.6	0.86
	Q18: Lack of proper facilities to discharge of emotions, has led to continued smoking.	0.762	0.6	0.80
	Q19: The media and social networks have an impact on continued smoking.	0.683	1	0.96
	Q20: Smoking by famous artists is an effective factor for adolescents to continued smoking.	0.645	0.6	1
	Q21: Not banning smoking in most places in the community (parks, coffee shops, etc.) has an effect on the continuation of smoking.	0.448	0.6	1
	Q22: The improper relationship between the parents and child/children has causes adolescents who have experienced cigarette smoking before, continue to smoke.	0.476	0.6	1
**F3: Cigarette dependence**	Q23: Addiction to smoking makes it difficult to quit.	0.422	0.8	1
	Q24: Due to dependence on cigarettes, the presence of a smoker in the group of non-smokers is tolerated.	0.431	1	0.90
	Q25: Dependence on smoking, reduces its physical and psychological risks.	0.702	0.8	0.93
	Q26: People who are addicted to cigarettes will smoke in any mood (happy or sad).	0.825	0.6	0.93
	Q27: People addicted to smoking, if they do not smoke one day, will suffer from nervous tension	0.800	1	0.96
	Q28: Cigarette addicts cannot do their daily activities well if they do not smoke for a day.	0.642	1	1
	Q29: Dependence on smoking, reduce social personality.	0.594	0.6	0.90
	Q30: Cigarette addiction restricts social activities (such as going out with family, attending in family gatherings, etc.)	0.544	0.8	0.90
	Q31: Addiction to smoking makes people always have cigarettes in their bags.	0.474	0.8	1
**F4: Intention to quit smoking**	Q32: With the increase in the price of cigarettes, adolescent smokers only change their type of cigarette and it has no effect on reducing their smoking.	0.582	0.6	0.78
	Q33: Maintaining a social personality is an obstacle to smoking anywhere.	0.778	1	0.86
	Q34: Having a purpose in life (such as educational and career goals, etc.) is a barrier to smoking.	0.819	0.8	0.93
	Q35: Choosing non-smoking friends reduces smoking in smokers	0.841	0.8	1
	Q36: Reducing the relationship with the smokers’ friends can reduces smoking.	0.800	0.6	1
	Q37: Anxiety and anger management skills are effective in reducing smoking.	0.739	0.6	0.93
	Q38: Choosing alternative behaviors (such as exercising, reading, watching movies, listening to music, etc.) can reduces smoking when upset and angry.	0.539	0.6	0.96
	Q39: Buying a cigarette instead of a pack of cigarettes can effectively reduce smoking.	0.527	0.6	1
	Q40: The skill of saying no to the suggestion of friends to smoke, reduces smoking.	0.490	0.8	0.96
**F5: Smoking cessation**	Q41: Having a non-smoking friend/friends encourages adolescents to quit smoking.	0.414	1	0.96
	Q42: The motivation to get better work and social opportunities in the future encourages smoking cessation.	0.659	0.6	0.80
	Q43: Comparing a smoker with a non-smoker can encourage a person to quit smoking.	0.558	0.6	0.80
	Q44: Encouraging important people in life’s person is effective in quit smoking.	0.687	0.6	0.93
	Q45: Negative reactions from others encourage smoking cessation.	0.706	0.8	0.90
	Q46: Watching videos and educational content about the dangers of smoking in the media (such as TV and social networks) can encourage adolescents to quit smoking.	0.646	0.8	1
	Q47: Appropriate alternatives to smoking (such as participating in sports, artistic activities, etc.) can effectively quit smoking.	0.693	0.8	1
	Q48: Training courses on the dangers of cigarette smoking in schools can encourage adolescents to quit smoking.	0.643	0.8	1
	Q49: Adolescents’ belief in their ability to control smoking can reduce or eliminate smoking.	0.620	0.6	0.78
	Q50: Observing the consequences of smoking in the people around can encourage that person to quit smoking.	0.537	1	0.93
	Q51: Rising cigarette prices will have a major impact on smoking cessation	0.479	0.8	1
	Q52: Feeling fear of tendency toward the use of other drugs or tobacco is effective in quitting smoking.	0.431	1	0.93

### Reliability

Based on the results of Cronbach’s alpha coefficients for 5 subscales of tendency to experience smoking, re-experience smoking, cigarette dependence, intention to quit smoking, and smoking cessation were 0.840, 0.844, 0.890, 0.849, and 0.867, respectively. The McDonald’s omega coefficients for the 5 subscales of the tendency to experience smoking, re-experience smoking, cigarette dependence, intention to quit smoking, and smoking cessation were 0.845, 0.849, 0.892, 0.854, and 0.871, respectively. Cronbach’s alpha and McDonald’s omega coefficients for all questions were 0.903 and 0.904, respectively. According to the results, the reliability of all 5 subscales and the entire questionnaire was acceptable (Table 3).

**Table 3:** Descriptive statistics of the subscales of questionnaire

Subscales	Item	Range	Cronbach’s alpha coefficients	McDonald’s omega coefficients
**F1: Tendency to experience smoking**	14	14–70	0.840	0.845
**F2: Re-experience smoking**	8	8–40	0.844	0.849
**F3: Cigarette dependence**	9	9–45	0.849	0.854
**F4: Intention to quit smoking**	9	9–45	0.890	0.892
**F5: Smoking cessation**	12	12–60	0.867	0.871
**Total CTQFA**	52	52–260	0.903	0.904

## Discussion

The purpose of this study was to design and evaluate the psychometric properties of the smoking tendency questionnaire for Iranian female adolescents. The results of this study showed that the instrument had acceptable validity and reliability. The validity of the questionnaire was evaluated using face validity, content validity, and construct validity. The CVR and CVI for all questions were 0.770 and 0.938, respectively. To perform the reliability of the instrument, Cronbach’s alpha coefficient, and McDonald’s omega coefficient were used, which were 0.903 and 0.904 for all questions, respectively. Finally, a questionnaire with 52 questions and 5 subscales of the tendency to experience smoking (14 items), re-experience smoking (8 items), cigarette dependence (9 items), intention to quit smoking (9 items) and smoking cessation (12 items) was approved. Based on the results, it is acceptable for CVR and CVI to be greater than 0.6 and 0.78 respectively, [37, 38]. Cronbach’s alpha value above 0.7 is acceptable and indicates strong internal consistency of the questions [45].

The first subscale of this questionnaire was “tendency to experience smoking”. This subscale was confirmed with 14 questions, CVR 0.6 to 1, CVI 0.83 to 1, regression coefficient (factor loading) 0.422 to 0.648, Cronbach’s alpha 0.840, and Omega McDonald 0.845. The tendency to experience smoking refers to “an individual’s inner desire and tendency to perform behavior primarily based on understanding the meanings of interpersonal and interpersonal communication”. Based on the Barati’s study, the results of the CFA showed that the belief-based tobacco smoking scale with 34-item and 4 subscales had an acceptable construct validity (RMSEA = 0.057; CFI = 0.933; IFI = 0.934; TLI = 0.918; GFI = 0.908 > 0.9). Also, Cronbach’s alpha of this questionnaire was between 0.55 to 0.92, CVI and CVR of the questionnaire were 0.89 and 0.80, respectively [46]. Based on the results of the Sterling study, the questionnaire of scales of smoking related self-efficacy, beliefs, and intention had acceptable construct validity (CFI = 0.96, NNFI = 0.96, RMSEA = 0.08) [28].

The second subscale of this questionnaire was “re-experience smoking”. This subscale was confirmed with 8 questions, CVR 0.6 to 1, CVI 0.80 to 1, regression coefficient (factor loading) 0.448 to 0.762, Cronbach’s alpha 0.844, and Omega McDonald 0.849. Re-experience smoking refers to “a person’s strong desire to perform a secondary behavior based on the experiences gained from performing the primary behavior”. In the Villalobos-Gallegos study, the results of CFA showed that the nicotine craving questionnaire with 12 items and three factors had acceptable construct validity. Also, the Cronbach’s alpha value of this questionnaire was between 0.86 and 0.90 for three factors, which indicated the reliability of the instrument [29]. Based on the results of the Dethier’s study, the construct validity of the on smoking urges questionnaire indicated that the questionnaire was confirmed by CFA (GFI = 0.984; AGFI = 0.972; PGFI = 0.569) [47]. The results of instrument reliability also showed that Cronbach’s alpha coefficient was 0.9 [47].

The third subscale of this questionnaire was “cigarette dependence”. This subscale was confirmed with 9 questions, CVR 0.6 to 1, CVI 0.80 to 1, regression coefficient (factor loading) 0.422 to 0.825, Cronbach’s alpha 0.849, and Omega McDonald 0.854. Cigarette dependence refers to “habits for doing behavior due to excessive repetition of behavior, physical and psychological dependence on performing the behavior”. Psychometrics of the cigarette dependence questionnaire by Etter showed that the correlation value of the test-retest was 0.83, Cronbach’s alpha was 0.84, and the instrument had acceptable reliability [30]. In the Morean study, Cronbach’s alpha for questionnaires of 22 questions, 8 questions, and 4 questions were 0.98, 0.93, and 0.86, respectively. Also, construct validity was verified by CFA (CFI > 0.90, RMSEA and SRMR < 0.08) [48].

The fourth subscale of this questionnaire was “intention to quit smoking”. This subscale was approved with 9 questions, CVR 0.6 to 1, CVI 0.78 to 1, regression coefficient (factor loading) 0.490 to 0.841, Cronbach’s alpha 0.890, and Omega McDonald 0.892. The intention to quit smoking refers to “motivation and inner desire to reduce behavior in the near future”. Based on the Blake’s study, the psychometric results of the smoking restraint questionnaire showed that the goodness of fit indices of the model had standard values (RMSEA =0.038, CFI = 0.99, TLI = 0.99) and the construct validity of the questionnaire was verified [31]. In Soleimani’s study, psychometric results of the situational temptation scale for smoking cessation with 9 questions showed that the CVI and CVR for each question were more than 0.71. The results of CFA showed that the construct validity of the questionnaire was acceptable (RMSEA = 0.006, GFI = 0.973, AGFI = 0.955). Also, Cronbach’s alpha was 0.80 and the reliability of the instrument was confirmed [49]. The results of the CFA in Can’s study showed that the questionnaire of challenges to stopping smoking with 21 items and two factors had the acceptable goodness of fit indices (RMSEA = 0.078, RMR = 0.011, CFI = 0.94, NFI = 0.90, IFI = 0.94). Also, the value of Cronbach’s alpha for two factors was 0.84 and 0.83, which indicated that the reliability of the questionnaire was acceptable [50].

The fifth subscale of this questionnaire was “smoking cessation”. This subscale was approved with 12 questions, CVR 0.6 to 1, CVI 0.78 to 1, regression coefficient (factor loading) 0.414 to 0.706, Cronbach’s alpha 0.867, and Omega McDonald 0.871. Smoking cessation refers to “the process of complete cessation of behavior”. In Liu’s study, the psychometric results of the smoking cessation counseling scale with 24 questions and four factors showed that this tool had good convergent validity. Also, Cronbach’s alpha was 0.95, which confirmed the reliability of the instrument [32]. Based on the psychometric results of the adolescent reasons for quitting smoking scale questionnaire, the 55 questions questionnaire in the CFA section explained 55% of the variance and had acceptable construct validity. Also, the results showed that Cronbach’s alpha of the questionnaire was between 0.72 and 0.87 and was confirmed [51].

One of the limitations of this study was that it coincides with the Coronavirus (COVID-19) pandemic, which makes the research process difficult. Another limitation of this study was the concern of schools and students’ parents to the subject of the study, which was resolved by providing explanations about study objectives.

## Conclusion

Due to the increasing prevalence of smoking among female adolescents, it is necessary to determine the reasons for their tendency to smoke, and design and implement appropriate educational programs in this field. This questionnaire with 52 questions and 5 subscales is a valid and reliable tool. The results of this study showed that the questionnaire of the tendency of female adolescents to cigarette smoking can be used as a valid tool to assess the current situation and evaluate programs related to smoking prevention in girls.

## Data Availability

The data generated or analyzed during this study is available from the corresponding author on reasonable request.

## Abbreviations

CVR: Content validity ratio
CVI: Content validity index
CFA: Confirmatory factor analysis
CTQFA: Cigarette smoking tendency questionnaire for female adolescents
× 2/df: Chi-square ratio to the degree of freedom
RMSEA: Root means the square error of approximation
RMR: Root means square residual
GFI: Goodness of fit index
PCFI: Parsimony comparative fit index
PNFI: Parsimonious normed fit index
PGFI: Parsimony goodness-of-fit index
CFI: Comparative fit index
